# Scaling of titanium implants entrains inflammation-induced osteolysis

**DOI:** 10.1038/srep39612

**Published:** 2017-01-06

**Authors:** Michal Eger, Nir Sterer, Tamar Liron, David Kohavi, Yankel Gabet

**Affiliations:** 1 Department of Anatomy & Anthropology, Sackler Faculty of Medicine, Tel Aviv University, Israel; 2 Department of Prosthodontics, Goldschleger School of Dental Medicine, Sackler Faculty of Medicine, Tel Aviv University, Israel

## Abstract

With millions of new dental and orthopedic implants inserted annually, periprosthetic osteolysis becomes a major concern. In dentistry, peri-implantitis management includes cleaning using ultrasonic scaling. We examined whether ultrasonic scaling releases titanium particles and induces inflammation and osteolysis. Titanium discs with machined, sandblasted/acid-etched and sandblasted surfaces were subjected to ultrasonic scaling and we physically and chemically characterized the released particles. These particles induced a severe inflammatory response in macrophages and stimulated osteoclastogenesis. The number of released particles and their chemical composition and nanotopography had a significant effect on the inflammatory response. Sandblasted surfaces released the highest number of particles with the greatest nanoroughness properties. Particles from sandblasted/acid-etched discs induced a milder inflammatory response than those from sandblasted discs but a stronger inflammatory response than those from machined discs. Titanium particles were then embedded in fibrin membranes placed on mouse calvariae for 5 weeks. Using micro-CT, we observed that particles from sandblasted discs induced more osteolysis than those from sandblasted/acid-etched discs. In summary, ultrasonic scaling of titanium implants releases particles in a surface type-dependent manner and may aggravate peri-implantitis. Future studies should assess whether surface roughening affects the extent of released wear particles and aseptic loosening of orthopedic implants.

The number of dental and orthopedic implant patients is increasing with an estimated 5 million new dental implants (www.persistencemarketresearch.com) and similar numbers of orthopedic prostheses each year worldwide. In dentistry, peri-implantitis is a recent but already major clinical concern and the main cause of long-term implant failure^1^,^2^,^3^. Peri-implantitis is triggered by specific oral bacteria and consists of inflammation that leads to bone resorption (osteolysis) around dental implants^4^. Once the peri-implant process starts, it can rarely be controlled and often results in implant loss^5^. Although there is no well-established treatment protocol for peri-implantitis, treatment usually begins with conservative attempts such as initiating mechanical cleaning of the surrounding biofilm using ultrasonic scaling and the use of antiseptic solutions and local and systemic antibiotic administration^2^,^3^. When these options fail to restrain disease progression, the implant is surgically removed.

The bond between a living bone and the implant surface is believed to be an important factor in clinical implant success. The factors contributing to osseointegration include implant composition, geometry and surface energy and texture^6^,^7^. Dental implant surfaces have been continuously modified, ranging from relatively smooth, machined (a.k.a. ‘turned’) surfaces to roughened surfaces. The roughening of implant surfaces is accomplished by physical procedures that involve blasting by various substances and/or acid etching^6^,^8^,^9^. Currently, virtually all commercially available implants undergo surface roughening. The majority of published reports show that there are benefits to using micro-roughening versus machined titanium (Ti) surfaces during the early healing stages of implant integration (up to three months)^6^,^7^. However, after three months, the use of rough implants presents no clear advantages^1^. Moreover, it is more challenging to prevent and manage peri-implantitis when using roughened implants instead of machined implants^1^.

Although Ti is considered to be a biocompatible material, long-term joint loosening after orthopedic joint replacement has been associated with aseptic inflammation. This inflammation is thought to be caused by the Ti particles that are released from the implant surface into the surrounding micro-environment, where they are phagocytosed by circulating monocytes and macrophages^10^,^11^. Macrophages engulf Ti particles causing them to secrete inflammatory cytokines, such as IL1β, IL6 and TNFα. These secreted cytokines are strong inducers of osteoclastogenesis and bone resorption^12^.

Here, we hypothesized that dental implant scaling, a procedure that is aimed at preventing peri-implantitis, may actually cause and aggravate the progression of the disease.

We therefore examined the release of Ti particles from several surface-treated Ti discs following ultrasonic scaling and determined the effects of these particles on inflammation and associated bone loss *in vitro* and *in vivo*. In these experiments, we aimed to characterize the factors that contribute to Ti particle-induced inflammation.

## Results

### Titanium Disc and Particle Characterization

#### Titanium Discs

To examine the topographic differences between Ti surfaces on machined (M), sand-blasted (SB) or sand-blasted and acid-etched (SLA) discs, we analyzed their nanoroughness using Atomic Force Microscopy (AFM, Table 1). Nanoroughness was characterized according to Z-range, Rq, Ra, R-max and surface area differentiation. The SB discs were found to have the greatest roughness out of all examined parameters, except surface area differentiation. SLA showed the second greatest values for all parameters, except surface area differentiation, for which it had the greatest value. The M surfaces displayed the lowest values for all roughness-related parameters. The XPS test for chemical composition revealed that SLA and SB surfaces were more contaminated than M surfaces by elements that were not related to the Ti alloy, such as Si, P, Ca and Zn (Table 2).

**Figure 1:**
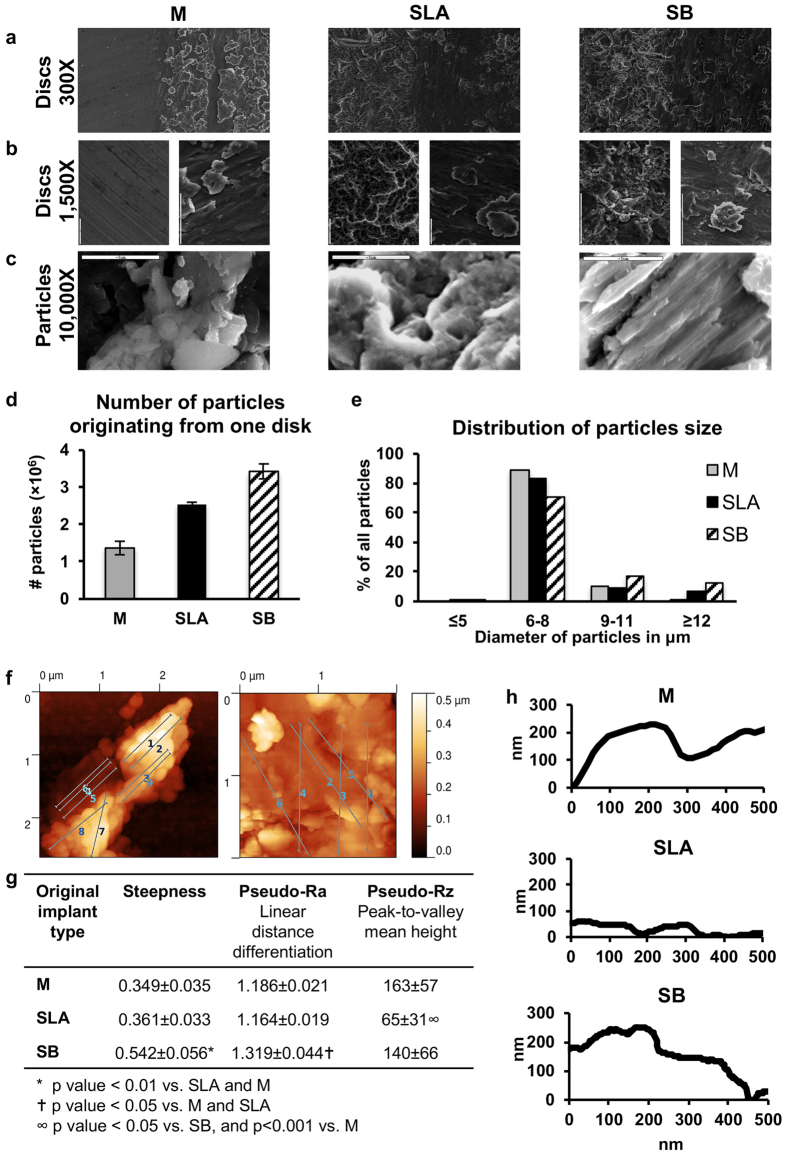
Topographic changes and titanium particles produced by US scaling. Three different titanium surfaces, including M, SLA, and SB, were treated with US scaling on half of the disc area. (a) The border was examined using SEM at ×300 magnification. (**b**) The areas on both sides of the midline were examined at ×1500 magnification. In each panel, the left side represents the original disc surface, while the right side shows the disc after treatment. (**c**) Titanium particles released during this process were visualized using SEM to estimate their micro-roughness, (**d**) the number of particles and (**e**) the size distribution of the particles, which were evaluated using an automated cell counter. The size distribution is shown as a percentage of the total number of particles that originated from each surface type. (**f**–**h**) Nanoroughness of particles was analyzed using atomic force microscopy (AFM). (**f**) Multiple line profiles were obtained for each particle type. (**g**) Slope, the fold-increase in linear distance and the average maximal distance between the highest and deepest points along 500 nm were calculated to determine particle nanoroughness. (**h**) Representative line profiles are shown for the different surfaces.

Half of the surface of each disc was scaled using a dental ultrasonic scaler, and the effect of scaling on each Ti surface was assessed using Scanning Electron Microscopy (SEM, Fig. 1a,b). As expected, scaling resulted in a smoother topography on the rough surfaces (SLA and SB). In contrast, the machined surface showed a relatively more irregular surface after scaling. Qualitatively, all 3 surface types, which were characterized by their very distinct topographies before scaling was performed, displayed a similar pattern after scaling.

#### Titanium Particles

We further assessed the differences between the Ti particles that were released from each surface. The numbers and size distributions of the particles that were released by scaling were evaluated using SEM and quantified using an automated Micro-Counter. The number of particles per surface area varied greatly among surfaces and was 48.5, 89.8 and 121.3 thousand particles/mm^2^ for the M, SLA, and SB discs, respectively (Fig. 1d). No significant difference was found in the sizes of the particles (7.57 ± 1.43, 7.57 ± 2.75 and 8.37 ± 2.94 μm for M, SLA and SB, respectively) or in their size distributions. The majority of the particles were within the 6- to 8-μm range (Fig. 1e). High-resolution SEM images (10,000× magnification) of the released particles suggested that there were differences in particle roughness (Fig. 1c). To confirm this finding, particle nanoroughness was examined using AFM-based line profilometry (Fig. 1f). Isolated particles were characterized for profile line steepness, linear distance differentiation (‘pseudo-Ra’) and peak-to-valley mean height (‘pseudo-Rz’). Steepness and linear distance differentiation were significantly higher for SB-derived particles, whereas the peak-to-valley mean height was significantly lower for SLA-derived particles (Fig. 1g). Energy-Dispersive X-ray spectroscopy (EDS) was used to evaluate the chemical composition of the particles (Table 2). Particles originating from all three Ti surfaces were contaminated with Cr, Fe, and Cu metal elements that were not found in the discs. To determine the origin of these metals, we analyzed the chemical composition of the ultrasonic tip that was used for disc scaling. All three metals, among others, were found. We therefore assumed that the contamination by Cr, Fe, and Cu originated from the scaler tip (Table 2).

Our data indicate that the numbers, nanoroughnesses and chemical profiles, but not the average sizes of the released particles, differed among the 3 surface types.

#### Inflammatory Response and Osteoclastogenesis Induced by Titanium Particles

**Figure 2:**
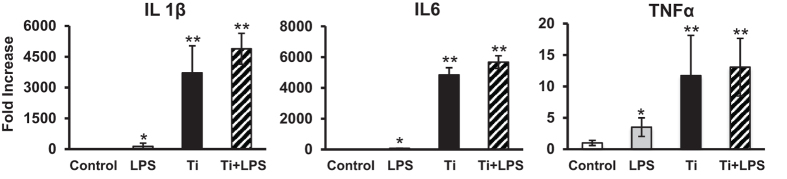
Titanium particles released from SLA titanium implants induce inflammatory responses in macrophages. BMDMs were cultured for 24 hours with titanium particles that were released by ultrasonic scaling (Ti) and/or bacterial LPS (control). Saline was used as the control. IL1β, IL6 and TNFα expression levels were measured using RT-qPCR, normalized to β-actin and expressed as a fold-change relative to the control level. The data are shown as the mean ± SD of n = 5 for each condition. These data are from a representative experiment out of 5 *p < 0.05 versus control; **p < 0.05 versus LPS and the control.

Ti particles have been repeatedly reported to induce an inflammatory response *in vivo* in the tissue surrounding implants and *in vitro*^11^,^13^,^14^. However, the pro-inflammatory response to the particles that originated from the ultrasonic scaling of dental implants has not yet been studied. Primary bone marrow-derived macrophages (BMDMs) were cultured for 24 hours with the Ti particles that were released by ultrasonic scaling of the SLA surface, which is the most common surface treatment that is used in commercially available dental implants. This setting is clinically relevant because it mimics the release of particles that occurs during routine cleaning around dental implant. Bacterial lipopolysaccharide (LPS) was added to a parallel set of cultures as a positive control^15^ and to Ti particles to assess additive/synergistic effects. The gene expression profiles of pro-inflammatory cytokines (IL1β, IL6, TNFα) indicated that the Ti particles induced a greater inflammatory response than 0.01 μg/ml LPS (a 40–70-fold increase, p < 0.001). The response to both LPS and Ti particles appeared to be additive rather than synergistic (Fig. 2). Because RNA expression does not always reflect secretion of these cytokines, we also analyzed protein levels of IL1β, IL6, TNFα in the supernatant of the macrophage cultures. Our multiplex analysis revealed a trend very similar to our RT-qPCR analysis with cytokines levels being the highest in the macrophages cultured with Ti particles (Fig. S1). To test whether the presence of Ti particles affects cell viability, we performed an apoptosis assay. We found that neither LPS nor Ti affected macrophage survival (Fig. S2).

**Figure 3:**
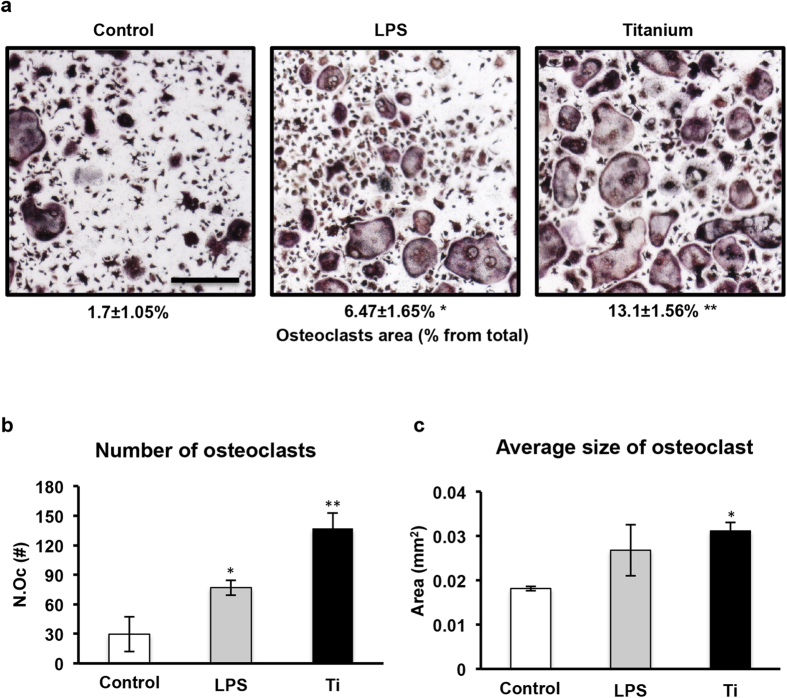
Titanium particles stimulate osteoclastogenesis *in vitro*. (**a**) TRAP staining (images and TRAP^+^area) of osteoclasts after 4 days of differentiation in the presence of LPS (0.01 μg/ml), titanium particles or diluent only (control). Bar = 30 μm. (**b**) The average number per well (N.Oc) and (**c**) the osteoclast area (mm^2^) in TRAP^+^cells after 4 days of differentiation. Data are shown as the mean ± SD. *p < 0.05 versus control; **p < 0.05 versus LPS and control.

To test the effect of titanium particles on osteoclast differentiation, we cultured pre-osteoclasts with LPS, SLA Ti particles or diluent alone in an osteoclastogenic medium. Notably, in the absence of RANKL, none of these conditions generated tartrate-resistant acid phosphatase (TRAP)+, multinucleated osteoclasts (Fig. S3). However, in the presence of RANKL, we were able to measure the effect of Ti particles on osteoclastogenesis. Similar to the inflammatory response, the presence of SLA particles significantly stimulated the size and total area of TRAP^+^cells to levels above those observed in both the control and LPS-treated cultures (Fig. 3, p < 0.005).

**Figure 4:**
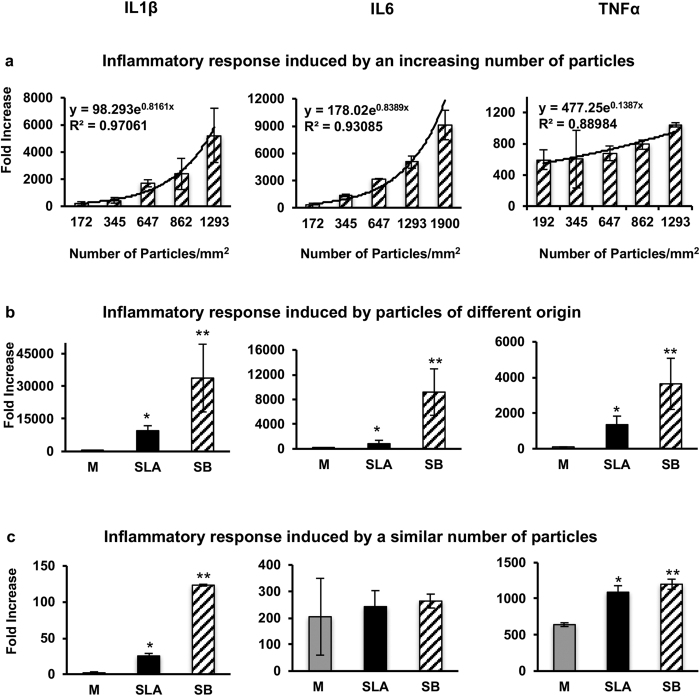
Inflammatory response dependent on the number and origin of titanium particles. BMDMs were cultured for 24 hours with: (**a**) Increasing numbers of titanium particles that were released by ultrasonic scaling of the SLA surface type. (**b**) 10% of the titanium particles that were released by ultrasonic scaling (Ti) of one disc from each implant surface: Machined (M), Sand-Blasted (SB), and SB/Acid-etched (SLA). (**c**) Same number of titanium particles (1293 particles/mm^2^) that were released by ultrasonic scaling (Ti) of each implant surface type: M, SB, SLA. IL1β, IL6 and TNFα expression levels were measured using RT-qPCR, normalized to β-actin and expressed as a fold-change relative to the control (no particles). The data are shown as the mean ± SD of n = 5 for each condition. These data are from a representative experiment out of 5. The regression analysis formula and goodness of fit (R^2^) are shown (a). *p < 0.05 versus M; **p < 0.05 versus SLA and M.

Based on our observation that scaling the different surfaces resulted in differences in the number of particles, we next assessed the effect of the number of particles on the inflammatory response. In these experiments, we cultured BMDMs with increasing numbers of particles that originated from SLA discs. Increasing the number of particles exponentially increased the inflammatory response, as indicated by ILβ, IL6, and TNFα expression levels (Fig. 4a). A regression analysis revealed that the inflammatory response (y) that was induced by the density of particles (x) could be predicted using the following formula:


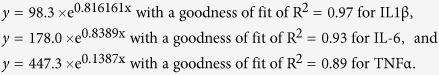


**Figure 5:**
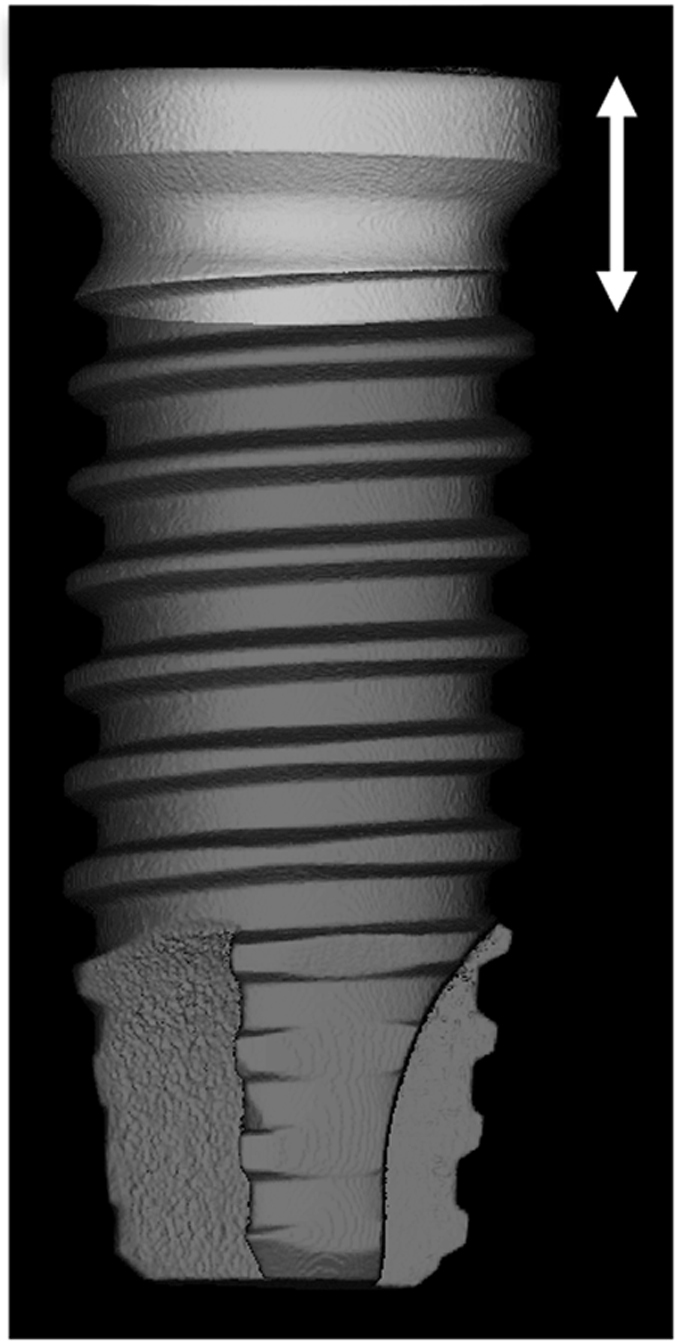
Schematic of exposed implant surfaces. Micro-CT 3D rendering of a 3.75 mm diameter implant with a putative horizontal bone loss of 2.05 mm (arrows). The resulting exposed region (light gray) corresponds to a total implant surface of 28.27 mm^2^.

To determine whether the extent of the inflammatory response is dependent upon the original implant surface, BMDMs were cultured with 10% of the particles that were released by scaling one disc (28.27 mm^2^ area) with each surface type, which is corresponded to 137 thousand M disc-produced particles, 254 thousand SLA disc-produced particles and 343 thousand SB disc-produced particles. The surface of the discs is equivalent to the exposed area in a 3.75-mm diameter implant following ~2 mm of vertical crestal bone resorption (Fig. 5). Each type of particle was placed in the well of a 6-well plate (9.6 cm^2^ area). An analysis of cytokine expression indicated that the particles originating from SB discs induced the most severe inflammatory response, whereas particles from machined surfaces induced the mildest response (Fig. 4b).

As mentioned above, there was no difference in the average size of the particles released from different implant types, but they did display distinct nanotopographies and chemical profiles. To assess whether the extent of the inflammatory response is dependent not only upon the number but also the type of original implant surface, BMDMs were cultured in 6-well plates with an equal number of particles, 1293 particles/mm^2^, from each surface type (M, SLA, or SB). This approach was aimed at nullifying the effect of particle number. The analysis of cytokine expression indicated that particles originating from SB discs induced the most severe inflammatory response, whereas the mildest response was observed in cells exposed to particles from machined surfaces (Fig. 4c). Overall, nanoroughness and/or chemical composition (which is dependent on the implant surface of origin) and particle number appear to have similar effects on the expression of pro-inflammatory cytokines in macrophages.

#### Titanium Particles Stimulate Inflammatory-Induced Osteolysis *in vivo*

**Figure 6:**
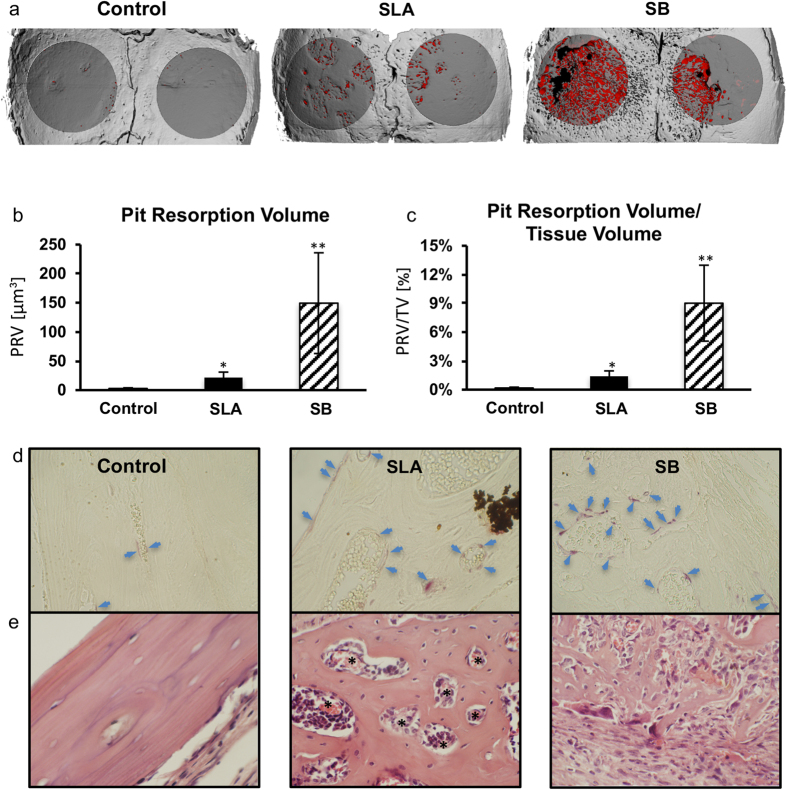
Titanium particles originating from US scaling of dental implants induce osteolysis *in vivo*. Titanium particles that originated from US scaling of SB and SLA discs were inserted into fibrin membranes and implanted onto the calvaria of 6 mice per group. Animals were then sacrificed after 5 weeks. (**a**) Representative μCT images of the calvaria are shown. The region of interest (ROI) is represented as dark gray, and the resorption pits are represented as red. (**b**) Pit Resorption Volume (PRV, μm^3^) and (**c**) PRV are shown relative to bone tissue volume inside the ROI (PRV/TV, %). The data are expressed as the mean ± SD, n = 6. *p < 0.05 versus control (membrane with no particles); **p < 0.05 versus SLA and control. (**d**) Histological TRAP-stained sections demonstrating the increase in lining osteoclasts (blue arrows). (**e**) HE staining demonstrating the presence of blood vessels (*), inflammatory cells and fibrous tissue, especially in the SB group. Original magnification ×40.

The effect of titanium particles on bone resorption was examined using a mouse calvaria model. Here we used 1.2 million particles for ‘SLA’ membranes and 1.8 million particles for ‘SB’ membranes. This number corresponds to the total number of particles released by US scaling of half a disc with SLA and SB surface respectively. In line with our *in vitro* results, 3D images reconstructed from the μCT data clearly showed that the surface of the parietal bone was eroded in the Ti particle groups (Fig. 6a). Pit resorption volume (PRV) and the pit resorption volume/tissue volume (PRV/TV) ratio in the ROI were measured. Quantitatively, particle-induced osteolysis was significantly higher in the titanium groups than in the sham controls. Moreover, bone loss was dramatically more severe in the SB group than in the SLA group (Fig. 6b,c). Histological analysis confirmed the increase in TRAP+ osteoclasts on the bone surface in response to SLA- and SB-originating Ti particles (Fig. 6d). It also further emphasized the dramatic bone resorption, especially in the SB groups, accompanied with the presence of inflammatory cells, fibrous tissue and new blood vessels (Fig. 6e).

## Discussion

In the present study, we examined the release of Ti particles following ultrasonic scaling and their effect on both inflammatory responses in macrophages and osteoclastic bone resorption. The magnitude of the inflammatory response was assessed in relation to specific variables, including the type of implant surface from which the particles originated in addition to particle size and number. Osteoclastogenesis and osteolysis were assessed both *in vitro* and *in vivo*.

The primary outcome of the study is that introducing the Ti particles that are produced by an ultrasonic metal tip to mouse BMDMs elicits an inflammatory response. The presence of Ti particles in BMDM cultures induced a significantly higher inflammatory response than was observed following exposure to LPS. Remarkably, adding LPS to cultures that already contained Ti particles did not significantly increase the inflammatory response. These data further emphasize the dominant pro-inflammatory influence of the Ti particles that are released by US scaling. The observed effect of Ti particles on osteoclastogenesis and bone loss was in line with the observed inflammatory response.

The secondary outcome of the study is that the physical properties of the particles differ according to the surface type from which they originate. The particles produced by US scaling differ in their nanotopographies, chemical profiles and numbers but not in their average sizes. Importantly, our data suggest that both the original implant type (which produces particles with different nanotopographies and chemical profiles) and the number of particles significantly contribute to the induction of an inflammatory response.

The deleterious side effects of the by-products that are released by US of Ti implants has not been previously studied. The results of our study are in accordance with the literature on innate immune responses to Ti particles, which have been extensively investigated, mainly with regard for the particles shed by orthopedic Ti prostheses^10^,^11^,^13^,^14^,^16^. In our study, US scaling of Ti surfaces induced the release of particles that stimulated the secretion of pro-inflammatory cytokines. An increase in the expression of IL1β, IL6 and TNFα, among other cytokines, was accompanied by an increase in osteoclast formation and activity, which was either directly or indirectly induced via a paracrine effect on neighboring cells (e.g. osteoblasts). These cytokines have been repeatedly shown to trigger and/or amplify inflammation-induced bone loss^13^. In addition, it was recently suggested that chronic stimulation by Ti particles might lead to a state of oxidative stress and persistent inflammation^17^.

A possible limitation of this study is that only short-term responses were assessed. We focused on acute responses to Ti particles and did not examine the long-term, chronic effects of exposure. However, the dramatic bone loss that was observed *in vivo* in the presence of Ti particles is unlikely to be reversible. Follow-up clinical studies are now needed to assess the long-term effects of US scaling of roughened Ti surfaces on particle-induced inflammation and osteolysis.

Another limitation is the number of particles that were introduced into the tissue culture. The particle number was produced by 60 seconds of scaling, which we considered to be a reasonable duration for cleaning a partially exposed implant. In clinical practice, a portion of the released particles would find their way outside the tissue. The expected number of released particles trapped in the surrounding tissues would therefore be less than the number we tested. However, Giovanni *et al*. studied inflammatory responses in macrophages that were induced by an ultra-low concentration of nanoparticles and found that pro-inflammatory cytokines were significantly stimulated^18^. Similarly, our own experiments, in which we used only 10% of the particles that were released from one disc, also resulted in a significant increase in IL1β and TNFα expression in macrophages, and a significant bone resorption was observed *in vivo* when the same number of particles was added to membranes in our calvaria model.

The present study also addresses the issue of mechanical cleaning of infected oral implants. Currently, only the effectiveness of bacterial biofilm removal and the damage to the implant surfaces has been described^19^,^20^. These changes depend on the initial topography of the implant and the type and mode of operation of the cleaning instruments^19^,^20^.

The results of the study confirm the hypothesis that ultrasonic-produced particles elicit a strong immune and osteolytic response. To the best of our knowledge, this report is the first study to describe the potential biological consequences of performing US scaling on Ti implants. Orthopedic implants also release particles, due to mechanical wear. Future research should elucidate whether surface roughening also affects the number and physical properties of these wear particles and assess their impact on inflammation and aseptic loosening of the prosthesis.

## Materials and Methods

### Titanium Disc Characterization

Ti discs were made from grade 23 Ti_6_Al_4_V, prepared as cylinders that were 6 mm in diameter (28.27 mm^2^ area) and 1.2 mm in height. The surface topography of the discs was machined (M), sand-blasted (SB) or sand-blasted and acid-etched (SLA) (AlphaBio Tec., Petah-Tikva, Israel). The surface of the discs is equivalent to the exposed area in a 3.75-mm diameter implant following ~2 mm of vertical bone loss (Fig. 1). The Ti discs were analyzed to determine their nanoroughness using atomic force microscopy (AFM, NanoWizard III, JPK, Berlin, Germany) and their chemical composition using X-Ray photoelectron spectroscopy (Scanning 5600 AES/XPS multi-technique system, PHI, USA) prior to scaling.

### Particle Generation and Characterization

Ti particles were generated using ultrasonic (US) scaling (Newtron Led, Satelec, Acteon, Marignac, France), adjusted to a frequency of 32 kHz. The resulting discs had one of the 3 previously described surface topographies. All particles were generated in a sterile environment. Each disc was submitted to US scaling for 60 seconds in distilled water (ddH_2_O) and then cleaned twice with ethanol and suspended in distilled water. The number of particles and their size distributions were evaluated using a Micro-Counter 1200 (Celeromics, Grenoble, France). Macro morphology was assessed using scanning electron microscopy (SEM, JSM-6300, JEOL Ltd, MA, USA) after the released particles were collected on carbon tape. An analysis of nanoroughness was performed using AFM. Because there is no established protocol for evaluating the nanoroughness of spherical particles, we used parameters similar to those utilized to analyze dental implant surfaces. Nine randomized line profiles were registered from particles originating from the 3 different types of surfaces. The average steepness was calculated for the x and y coordinates of the line profile every 10 nm along a linear distance of 500 nm. Linear distance differentiation (similar to Ra/surface area differentiation) was calculated using the Pythagorean formula to assess the increase between the actual line profile and the straight linear distance over 500 nm (one-dimensional measure). Peak-to-valley mean height (similar to Rz) was calculated as the average height difference between the highest peak and the deepest valley along the recorded 500 nm. The chemical composition of the particles was analyzed using energy-dispersive X-ray spectroscopy (EDS, JSM-6300, JEOL Ltd, MA, USA).

### Cell Culture

All procedures involving animals were carried out in accordance with the guidelines of the Tel Aviv University and were approved by the Institutional Animal Care and Use Committee (permit number M-015–047).

Primary bone marrow-derived macrophages (BMDMs) were isolated from the femora and tibiae of adult C57BL/6J-RCC mice, as previously described^21^. Briefly, cells were cultured overnight in 6-well dishes at 37 °C in a humidified atmosphere with 5% CO_2_ in alpha modified Eagle’s medium (αMEM, Life Science Technology, NY, USA). After 24 hours, the non-adherent fraction was cultured in 10-cm non-culture-treated dishes containing αMEM supplemented with 10% fetal bovine serum (FBS, Rhenium Ltd, Modi’in, Israel) and 100 ng/ml macrophage colony stimulating factor (M-CSF). M-CSF was obtained from CMG (14–12) cells as previously described^22^. The resulting adherent BMDMs were collected and seeded in 6-well plates (10^6^ cells/well). Ti particles with different origins, bacterial lipopolysaccharide (LPS, 0.01 μg/ml, used as the positive control), or diluent only (control) were added to the cultures, and the cells were incubated for 24 to 48 hours as indicated. This dose of LPS has been reported to induce a significant inflammatory response in primary macrophages^15^.

For the osteoclastogenesis assay, BMDM, which are the same as preosteoclasts *in vitro*, were plated in 96-well plates (7,000 cells per well) in standard medium supplemented with 20 ng/ml M-CSF and 50 ng/ml RANKL (R&D Systems, Minneapolis, MN, USA), replaced every 2 days, as previously described^21^. Treatments (either LPS or titanium particles) were added after two days of incubation. On the 4^th^ day, cells were stained using a TRAP kit (Sigma-Aldrich, St. Louis, MO, USA), and multinucleated (<3 nuclei) TRAP-positive cells were defined as osteoclasts. Images were acquired at an original magnification of × 4 (Evos FLC, Life Technologies, MS, USA). Osteoclast number and total osteoclast area were measured using ImageJ software (National Institutes of Health, Bethesda, MD, USA).

### Apoptosis

For analysis of apoptosis, macrophages were harvested by trypsinization 24 hours after medium change, and stained with FITC-conjugated Annexin V and propidium iodide for 15 min, according to the manufacturer’s instructions (MBL, Nagoya, Japan). Cells positive for Annexin V and propidium iodide were recorded using flow cytometry (Gallios, Beckman Coulter, Indianapolis, IN, USA). The results were analyzed using Kaluza software (Beckman Coulter, Indianapolis, IN, USA).

### Protein analysis, RNA Isolation and RT-qPCR

After incubation, the supernatant was collected and secreted protein amounts of IL1β, IL6 and TNFα were measured using multiplex assay and expressed in MFI units (Multiplex Fluorescent Immunoassay, ProcartaPlex Multiplex Immunoassay, eBioscience, San Diego, CA, USA). After supernatant collections, macrophages were washed with sterile PBS, and RNA was extracted using Tri-RNA Reagent (Favorgen Biotech Corp, Kaohsiung, Taiwan). The 260/280 absorbance ratio was measured to verify RNA purity and concentration. cDNA was produced using a High Capacity cDNA Reverse Transcription Kit (Invitrogen, Grand Island, NY, USA), and real-time PCR was performed using Kapa SYBR Fast qPCR (Kapa Biosystems, Wilmington, MA, USA) on a StepOne real time PCR machine (Applied Biosystems, Grand Island, NY, USA). We examined the expression of IL1β, IL6 and TNFα, which are established markers of macrophage inflammation. The primer sets were as follows: F-GAAATGCCACCTTTTGACAGTG and R-TGGATGCTCTCATCAGGACAG for mouse IL1β; F-TAGTCCTTCCTACCCCAATTTCC and R-TTGGTCCTTAGCCACTCCTTC for mouse IL6; and F-TCTTCTCATTCCTGCTTGTGG and R-GGTCTGGGCCATAGAACTGA for mouse TNFα. The reaction was subjected to 40 cycles of amplification using the following program: 95 °C for 20 s, 60 °C for 20 s, and 72 °C for 25 s. The relative mRNA expression levels of the selected genes were normalized to the level of β-actin, which was amplified using the following primers: F-GTCACCCACACTGTGCCCATC and R-CCGTCAGGCAGCTCATAGCTC.

### Animal Model and Micro-Computed Tomography (μCT)

#### Membrane preparation

Titanium particles (generated as described before) were embedded in fibrin membranes, which were used as scaffolds to localize the titanium particles. Fibrin membranes were prepared in 48-well plates by mixing fibrinogen from bovine plasma with thrombin from bovine plasma (Sigma-Aldrich, St. Louis, MO, USA). Membranes with no particles were prepared as controls.

#### Surgical insertion model

After anesthesia, the skin of C57Bl/6J-Rcc female mice was shaved and disinfected at 10 weeks of age. The parietal bones of the mice were exposed via a 10-mm incision in the nape area, and the periosteum was removed using a periosteal elevator. Membranes with titanium particles with different surfaces were inserted to cover both parietal bones. The surgical incision was then closed using nylon monofilament surgical sutures (5/0). In the sham controls, membranes with no particles were inserted and the incisions were closed. All groups consisted of 6 animals.

After a follow-up period of 5 weeks, the animals were sacrificed and the skull of each mouse was removed, fixed for 24 hrs in 4% phosphate-buffered formalin followed by ethanol 70%. All specimens were scanned and analyzed using a μCT system (μCT 50, Scanco Medical AG, Switzerland). Scans were performed at a 10-μm resolution in all three spatial dimensions, with 90 kV energy, 88 μA intensity and 1000 projections at a 1000 msec integration time. The region of interest (ROI) was defined as two 3.7-mm circles in the center of the parietal bones. The mineralized tissues were differentially segmented using a global thresholding procedure^23^. A custom-made algorithm based on Image-Processing Language (IPL, Scanco Medical) was developed to isolate the resorption pits, defined as unmineralized pits that were 10- to 40-μm deep on the bone surface. The measured resorption volume was limited to a 40 μm depth because beyond that, the resorption pit was connected to the internal diploe. Morphometric parameters were determined using a direct 3D approach^24^ and included the total volume of the bone resorption (Pit Resorption Volume, PRV, μm^3^) and bone tissue volume inside the ROI (TV, μm^3^), which was used to determine the PRV/TV (%).

### Histological analysis

Following μCT scanning, the specimens underwent decalcification in 10% EDTA, dehydration in graded alcohols and embedding in paraffin. Each calvaria was serially sectioned to a thickness of 5 μm in the coronal plane. Each section was sampled three times, 0.5 mm apart. Half the sections were stained with hematoxylin-eosin (HE) and serial sections with tartrate-resistant acid phosphate (TRAP, Sigma). Images were acquired using an Olympus BH2 microscope (Olympus, Japan), attached to an Olympus DP70 camera. All images were acquired using a ×40 lens and further enlarged 10-fold by the camera (final magnification ×400).

### Statistical analysis

Values are expressed as the mean ± SD unless otherwise indicated. Statistical analyses were performed using GraphPad Prism 7.0 (La Jolla, CA, USA). As all presented data typically display a normal distribution, analysis of variance (ANOVA) and Tukey’s post hoc test for multiple group comparison were used. Differences between groups were defined as significant at *p* < 0.05.

### Summary

Ti particles were introduced to BMDMs by applying an ultrasonic metal tip to titanium discs. This elicited an inflammatory response and resulted in dramatic bone loss. The Ti particles induced a significantly higher inflammatory and osteoclastogenic response than was induced by LPS. The physical properties of the particles that were derived from different implant surface types differed in their nanotopographies, chemical compositions and numbers but not their average sizes. The implant surface type and the number of particles significantly contributed to the induction of inflammation. This study suggests that the biological consequences of implant cleaning procedures have a significant clinical impact. Thus, evaluations of treatment results should concentrate not only on the efficiency of the cleaning and the damage caused to the implant surface but also on the quantity and properties of the released particles.

## Additional Information

**How to cite this article:** Eger, M. *et al*. Scaling of titanium implants entrains inflammation-induced osteolysis. *Sci. Rep.* **7**, 39612; doi: 10.1038/srep39612 (2017).

**Publisher's note:** Springer Nature remains neutral with regard to jurisdictional claims in published maps and institutional affiliations.

## Supplementary Material

Supplementary Information

## Figures and Tables

**Table 1:** Nanoroughness characterization of M, SLA and SB titanium discs, measured using AFM.

	M	SLA	SB
**Z range (nm)**	670.00	4049.17	4414.67
**Rq (nm)**	100.15	680.83	763.33
**Ra (nm)**	80.40	558.33	617.83
**R max (nm)**	661.33	3963.50	4421.33
**Surface area differentiation (ratio)**	0.03	0.34	0.27

**Table 2:** Chemical characterization of M, SLA and SB titanium discs and particles.

	Titanium Discs (XPS)			Titanium Particles (EDS)			US Tip (EDS)
	M	SLA	SB	M	SLA	SB	—
**C**	33.04	26.65	25.00	19.65	28.622	25.74	22.49
**O**	47.25	49.96	48.82	15.17	23.30	28.63	ND
**Ti**	13.02	15.94	9.47	56.82	40.25	33.19	ND
**Al**	2.71	2.91	13.22	4.89	3.28	4.9	0.55
**V**	0.25	0.33	0.12	2.77	2.01	1.70	ND
**N**	2.82	2.95	1.18	ND	ND	ND	ND
**S**	0.55	0.62	0.39	ND	ND	ND	ND
**Cl**	0.16	ND	0.06	ND	ND	ND	ND
**Pb**	0.21	0.01	ND	ND	ND	ND	ND
**Si**	ND	0.16	0.75	ND	ND	ND	0.17
**P**	ND	0.09	ND	ND	ND	ND	ND
**Ca**	ND	0.04	0.22	ND	ND	ND	0.05
**Zn**	ND	0.33	0.08	ND	ND	ND	ND
**Cr**	ND	ND	ND	0.05	0.35	0.04	13.45
**Fe**	ND	ND	ND	0.61	2.13	0.44	64.24
**Cu**	ND	ND	ND	0.04	0.048	0.12	1.78
**Ni**	ND	ND	ND	ND	ND	ND	1.63
**F**	ND	ND	ND	ND	ND	ND	2.36

The chemical profiles of sterilized titanium discs were analyzed using XPS (elements determined according to the Mendeleev periodic table). The titanium particles that were released by US scaling and the US tip were analyzed using EDS.
