# Supplementary material for: Scaling of titanium implants entrains inflammation-induced osteolysis
Source: Sci Rep. 2017 Jan 6;7:39612. doi: 10.1038/srep39612 (PMC5216395; doi:10.1038/srep39612)
Supplement: Supplementary Information [file srep39612-s1.pdf]

Scaling of titanium implants entrains inflammation-induced osteolysis

Eger Michal[1], Sterer Nir[2], Liron Tamar[1], Kohavi David[2], Gabet Yankel[1*]

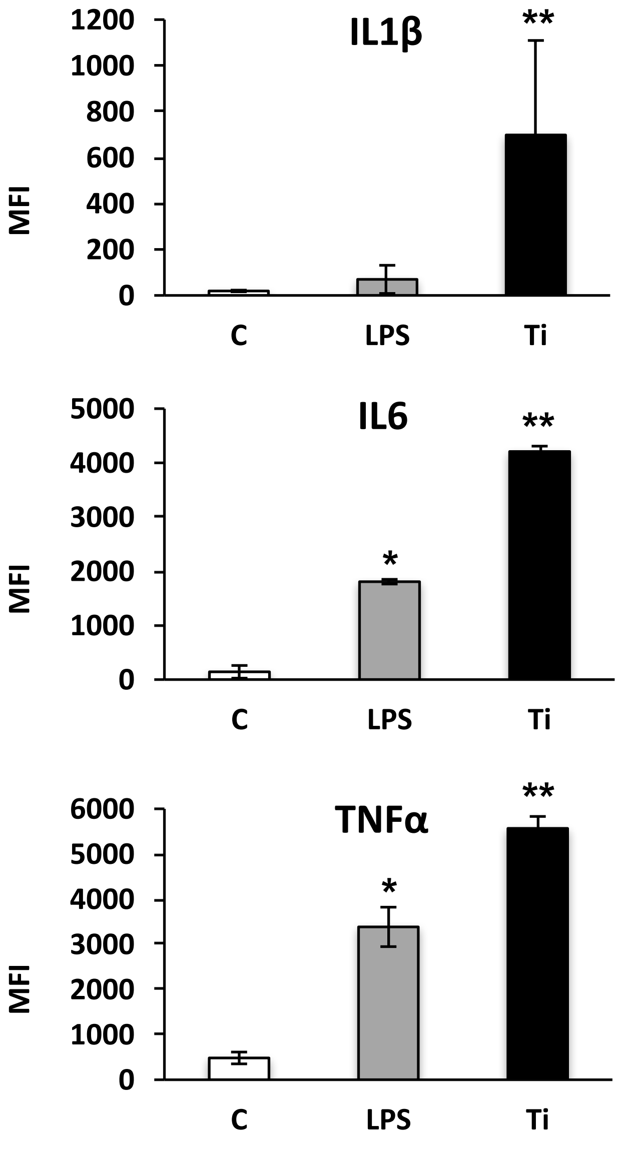

Figure S1. Titanium particles released from SLA surface induce the secretion of inflammatory cytokines. BMDM were cultured for 24 hours with titanium particles that were released by ultrasonic scaling (Ti), or bacterial LPS (LPS). Saline was used as control (C). Secreted protein amounts of IL1β, IL6 and TNFα were measured in the supernatant using multiplex assay and expressed in MFI units (Multiplex Fluorescent Immunoassay).

Data are shown as the mean±SD from 3 independent experiments performed in triplicates. *, $p<0.005$ versus control; **, $p<0.005$ versus LPS and the control.

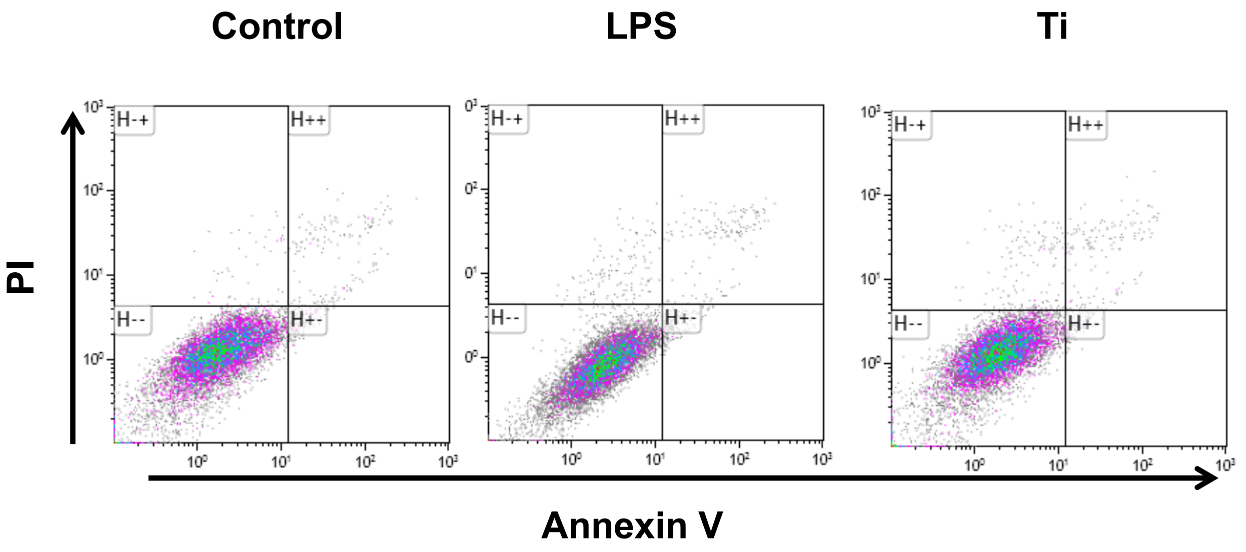

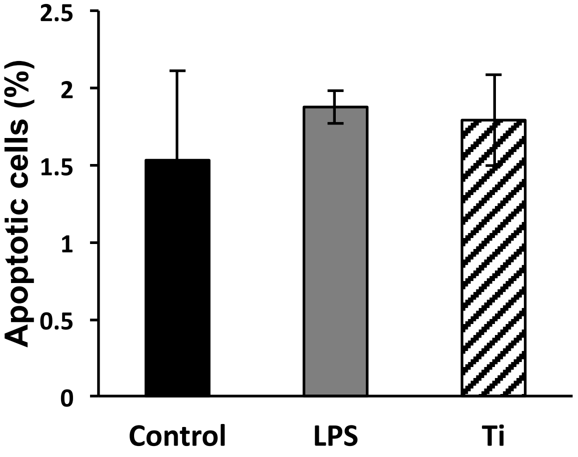

Figure S2. Titanium particles do not induce apoptosis in macrophages. BMDM were cultured for 24 hours with titanium particles that were released by ultrasonic scaling of SLA discs (Ti), or bacterial LPS (LPS). Saline was used as control. Apoptosis was measured using flow cytometry based on Annexin V and PI staining. Data are shown as the mean±SD of 6 independent experiments from 6 mice, each performed in triplicates. Ti *vs*. Control

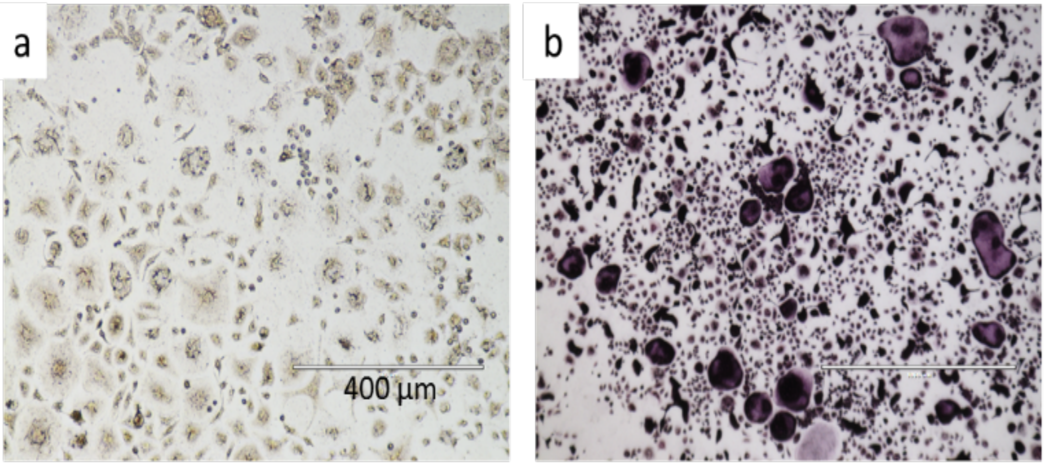

Figure S3. LPS did not induce osteoclastogenesis in the absence of RANKL. (a) Osteoclastogenesis and TRAP staining were performed as in Fig. 3, in the presence of M-CSF and LPS (0.01 µg/ml), without (A) or with 50 ng/ml RANKL (B). Bar = 400 µm.
